# A Scalable Framework to Detect Personal Health Mentions on Twitter

**DOI:** 10.2196/jmir.4305

**Published:** 2015-06-05

**Authors:** Zhijun Yin, Daniel Fabbri, S Trent Rosenbloom, Bradley Malin

**Affiliations:** ^1^ Dept. of Electrical Engineering & Computer Science Vanderbilt University Nashville, TN United States; ^2^ Dept. of Biomedical Informatics Vanderbilt University Nashville, TN United States; ^3^ Dept. of Medicine Vanderbilt Univerisity Nashville, TN United States; ^4^ School of Nursing Vanderbilt University Nashville, TN United States; ^5^ Dept. of Pediatrics Vanderbilt University Nashville, TN United States

**Keywords:** consumer health, information retrieval, machine learning, social media, twitter, infodemiology

## Abstract

**Background:**

Biomedical research has traditionally been conducted via surveys and the analysis of medical records. However, these resources are limited in their content, such that non-traditional domains (eg, online forums and social media) have an opportunity to supplement the view of an individual’s health.

**Objective:**

The objective of this study was to develop a scalable framework to detect personal health status mentions on Twitter and assess the extent to which such information is disclosed.

**Methods:**

We collected more than 250 million tweets via the Twitter streaming API over a 2-month period in 2014. The corpus was filtered down to approximately 250,000 tweets, stratified across 34 high-impact health issues, based on guidance from the Medical Expenditure Panel Survey. We created a labeled corpus of several thousand tweets via a survey, administered over Amazon Mechanical Turk, that documents when terms correspond to mentions of personal health issues or an alternative (eg, a metaphor). We engineered a scalable classifier for personal health mentions via feature selection and assessed its potential over the health issues. We further investigated the utility of the tweets by determining the extent to which Twitter users disclose personal health status.

**Results:**

Our investigation yielded several notable findings. First, we find that tweets from a small subset of the health issues can train a scalable classifier to detect health mentions. Specifically, training on 2000 tweets from four health issues (cancer, depression, hypertension, and leukemia) yielded a classifier with precision of 0.77 on all 34 health issues. Second, Twitter users disclosed personal health status for all health issues. Notably, personal health status was disclosed over 50% of the time for 11 out of 34 (33%) investigated health issues. Third, the disclosure rate was dependent on the health issue in a statistically significant manner (*P*<.001). For instance, more than 80% of the tweets about migraines (83/100) and allergies (85/100) communicated personal health status, while only around 10% of the tweets about obesity (13/100) and heart attack (12/100) did so. Fourth, the likelihood that people disclose their own versus other people’s health status was dependent on health issue in a statistically significant manner as well (*P*<.001). For example, 69% (69/100) of the insomnia tweets disclosed the author’s status, while only 1% (1/100) disclosed another person’s status. By contrast, 1% (1/100) of the Down syndrome tweets disclosed the author’s status, while 21% (21/100) disclosed another person’s status.

**Conclusions:**

It is possible to automatically detect personal health status mentions on Twitter in a scalable manner. These mentions correspond to the health issues of the Twitter users themselves, but also other individuals. Though this study did not investigate the veracity of such statements, we anticipate such information may be useful in supplementing traditional health-related sources for research purposes.

##  Introduction

### Background

Traditional methods for collecting data in support of clinical research include prospectively collected surveys (eg, [1]), retrospective analyses of existing medical records (eg, [2,3]), and a combination of the two (eg, [4]). Over the past decade, computerized methods for data collection have emerged, with traditional surveys for health research moving onto the Internet [5] and increasingly widespread electronic medical records (EMRs) able to be mined to investigate a wide range of acute and longitudinal phenotypes [6-8]. At the same time, these approaches tend to focus only on a medically centric worldview, and may provide only a partial view of a patient’s life. Recognizing this limitation, investigators have suggested that the data contributed through non-traditional domains, such as mobile apps [9-11] and online forums where patients self-report on their status [12,13], will provide a more complete view of an individual’s health and population-based health trends.

An increasing number of studies demonstrate that the data disseminated via social media platforms, such as Twitter, can inform health-related investigations. We review such studies in the following section, but we highlight that studies have shown, for instance, that such data can be mined to model aggregate trends about health (eg, detection of statistically significant adverse effects of pharmaceuticals [14,15]). Recent investigations have also demonstrated that an individual’s health status can be corroborated by the statements they publish over social media platforms (eg, confirmation of flu diagnoses [16]). Despite the power of such investigations, they are limited in that the associated approaches do not filter data from social media streams for any arbitrary health-related concept.

### Objective and Contribution

The objective of our work is to develop a scalable framework for detecting mentions about personal health on a specific social media platform, namely Twitter. The system introduced in this paper is composed of several core processes. First, the system filters the Twitter stream for tweets that are likely to contain health-related information. Next, a subset of the tweets are labeled with respect to the type of information that is communicated (eg, health status of the author versus a metaphorical statement) and applied to train a classifier. While it is possible to label a large number of tweets given a substantial budget, it is unlikely that a classifier could be specialized for each specific health issue. For instance, imagine a researcher is interested in studying 10,000 distinct health issues, each of which will require at least 500 tweets to train a robust classifier. If the cost to label each tweet is $0.10, it would cost $500,000 to build the necessary corpora! Our framework demonstrates that a scalable classifier, which discovers health mentions across a broad range of health issues, can be composed by leveraging a mixture of tweets from various health issues, which could make large-scale investigations much more cost-effective. In doing so, however, our system is oriented toward a high precision while maintaining a reasonable recall.

There are three primary contributions of this paper:

- Labeled Health Mention Corpus. We leverage Amazon Mechanical Turk to create a labeled corpus of tweets with health mentions for 34 health issues. These include certain high impact health issues investigated in the Medical Expenditure Panel Survey [17], such as arthritis, asthma, bronchitis, cancer, diabetes, hypertension, and stroke.
- Health Mention Detection. We introduce a system to automatically detect personal health mentions in tweet streams. We show that this system is trainable with a relatively small number of labeled tweets from several health issues. Moreover, it can effectively detect personal health mentions across a range of health issues on Twitter. For instance, training on 2000 tweets associated with four health issues (cancer, depression, hypertension, and leukemia) can yield a classifier that achieves a precision of 0.77 on the aforementioned corpus of tweets of 34 health issues.
- Health Mention Attribution. To demonstrate the potential for the data filtered from Twitter, we investigated how people reveal information about themselves and others. In doing so, we show that the likelihood an individual self-discloses is dependent on the health issues communicated. For example, personal health status is revealed more than 50% for 11 of the 34 health issues. For certain health issues (eg, allergies, bronchitis, insomnia, migraines, and ulcers), people are more likely to disclose their own health status, while for other health issues (eg, Alzheimer’s, Down syndrome, leukemia, miscarriage, and Parkinson’s), people are more likely to disclose another person’s status.

### Prior Work

#### Social Media and Health Research

As alluded to, various investigations have demonstrated that social media can be successfully leveraged to (1) enable individuals to discuss their health status, (2) influence an individual’s health behavior, and (3) support the analysis of aggregate trends around health activities.

First, a certain portion of studies have focused on the extent to which, as well as how, social media enables self-reports of health information. Hale et al [18] showed that users discuss their health conditions on public Facebook pages, but recognized that such pages tend to be overly general to attract users to contribute to a discussion. However, Bodnar and colleagues [16] found that individuals who use social media discuss certain ailments with high accuracy on Twitter. Specifically, it was demonstrated that college students tend to talk about their influenza diagnosis and associated symptoms. More generally, Paul et al [19] performed latent topic model discovery over self-reported health status in Twitter to detect complex and potentially novel phenotypes. It has further been shown, that some Twitter users reveal genome sequencing results (in relation to ancestry information according to 23andme.com services) over Twitter [20].

Second, the previous investigations show that individuals publish information about themselves, but there is also a growing body of evidence to suggest that social media can influence an individual’s health behavior. In certain cases, exploitation of social media can bring about negative health behaviors. For instance, based on discussions about prescription abuse over Twitter, it was observed that social media may aggravate such problems [21,22]. In a similar vein, a content analysis of tweets, in association with the demographics of the followers of marijuana Twitter handles, showed that social media may allure young people to establish substance use patterns. Wilson et al also argued that social media enables more individuals to be involved in an anti-vaccination movement [23]. However, it was also shown that social media can encourage more positive changes in health behavior. Notably, it was shown that increasing communications with smokers on social media can promote free cessation services [24]. Moreover, Cobb and colleagues [25] developed a Facebook application that was able to track the significant elements of an intervention on smoke cessation. It was also found that the design and realization of a community opinion leader model may mitigate the spread of HIV [26].

Third, social media can be mined to learn and characterize aggregate trends with respect to health activities. For instance, it was shown that flu trends can be effectively extracted from Twitter using standard machine learning strategies [27]. More specifically, the analysis of daily tweets across a major metropolitan region (eg, New York) can enable the prediction of which health issues are currently influencing the health of the public [28]. Meanwhile, Nagel et al [29] showed that both the keywords chosen to filter and create subgroups of tweets affected prediction accuracy. Beyond health status, it has been illustrated that the rare or unknown side-effects of drugs can be discovered through sentiment analysis over Twitter [15].

Though social media can support a wide array of health-related investigations, there are a number of hurdles to making the associated methodologies scalable. As Curtis and colleagues [30] point out, for instance, insufficient procedures for protecting participants’ privacy was one of the challenges to recruiting members from social media to conduct HIV research. In addition, it was recently revealed that the unreliability of big data and continuous changes of search algorithms contributed to failures in the Google Flu Trends program [31].

Our work differs from the aforementioned studies in that we focus on personal health status disclosure on Twitter. We note that Mao et al [32] discussed a similar topic, but their work is limited in that (1) it relied on regular expressions for classification, (2) focused on a limited number of health issues, and (3) examined whether personal health status is disclosed on status or conversation, but did not differentiate when heath status was disclosed for authors versus others. Lamb et al [33] showed that a combination of tweets about infection with respect to both authors and others performed better than tweets about the authors alone when predicting flu trends, which lends credibility to our work. However, it should be noted that their classification only focused on a diagnosis of the flu instead of a broad range of health issues, as is addressed in our work.

#### Classification on Social Media

To mine health-related information from social media, it is critical to develop a classifier. However, tweets are constrained in size and, thus, are composed of limited content. Consequentially, it is essential to define and select discriminative features to support automated health status detection. In certain studies, tweets were enriched with features by referencing external sources, such as Wikipedia [34,35], to improve topic modeling, but their generality hampers them in the support of personal health mention detection.

As an alternative, it has been shown that punctuation, emoji characters, hashtags, and the @username designation, as well as text (including n-grams of words or characters [36]) from the webpage referenced by the URL in a tweet, can form meaningful features for classification purposes [34,37,38]. Features generated using natural language processing tools, such as part of speech tags and dependencies between terms were also successfully incorporated as features in social media classifiers [33,39]. Building on previous studies, our work illustrates that nouns, verbs, pronouns, punctuation, emoji, hashtags, as well as dependencies, can serve as effective features for personal health mention.

#### Social Media Corpus Construction

If we rely on a classifier to filter and analyze social media, then it is essential to obtain (or create) a labeled corpus to train the classifier. Crowdsourcing over Web-based platforms, such as Amazon Mechanical Turk (MT), has been employed to generate labeled gold standard corpora [37]. Notably, MT was leveraged to label when tweets were related to the health status of the author of a tweet in the latent topic modeling analysis discussed above [19]. However, it should be recognized that the survey utilized by [19] is limited in that it only related tweet content to the author and not another person’s health status.

### The Personal Health Status Mention Problem

To formalize the problem, we define the notions of personal health status and mention: Definition 1 (Personal Health Status) is the health condition of a specific person regarding a health issue or symptom, and Definition 2 (Personal Health Mention) is a statement of personal health status in social media.

These definitions focus on the health information of the individuals who are potentially identifiable. For instance, tweets such as “my father is cancer free for ten years”, “I have to do chemo tomorrow”, and “my little cousin has leukemia” are representatives of personal health mentions. By contrast, “Local charity doing great work to help cancer patients” is not a personal health mention because the subject is a group of people as opposed to a specific person.

We treat the problem of personal health mention detection as binary classification. We say a tweet is positive if it reveals personal health status and negative otherwise. For example, two MT masters assigned positive labels to each of the first three tweets in Table 1 (details in Method Section). Yet a term associated with a health issue can be uttered on Twitter for many other reasons, such as in a metaphorical sense, to express a viewpoint about a health issue in general, or to communicate a worry. The next three tweets in Table 1 provide examples of these reasons respectively.

Given their brevity (140 characters at most), tweets often have limited context. Consequentially, assigning a class label to a tweet is substantially more challenging than detecting if a given tweet communicates status of the author. The last three tweets in Table 1 illustrates this observation, where MT masters assigned different option labels to the same tweet.

In this paper, we study how people disclose personal health statuses on Twitter and present a scalable personal health mentions detection system for the Twitter stream. Specifically, we decompose this investigation into the following four hypotheses: H1: People discuss personal health status on Twitter; H2: Personal health status disclosure rate is health issue dependent; H3: The likelihood that people disclose their own versus other people’s personal health status is health issue dependent; and H4: Personal health status mention classifiers based on tweets of multiple health issues are more scalable than those based on a single health issue.

**Table 1:** Examples of tweets related to health issues and the labels obtained through the Mechanical Turk (MT) survey.

Tweet		Label via MT	
		Master 1	Master 2
**Positive**			
	I’m suffering from schizophrenia and a little bit of insomnia.	author	author
	Prayers for my dad would be appreciated. He has lymphoma. Thanks for the support everyone.	relative	relative
	didn’t she have a miscarriage like 3 days ago?	someone else	someone else
**Negative**			
	you’re gonna give Viv a heart attack	metaphor	metaphor
	Even after Bill Gates relentless support and millions of dollars poured into Malaria research, we are not successful.	viewpoint	viewpoint
	Praying I don’t have pneumonia	worry	worry
**Ambiguous**			
	Cheerios say she’ll never have to worry about dieting. Too bad with 2:1 sodium to cal, she’ll have to worry about high blood pressure.	metaphor	someone else
	Yooo soo i walk out my apt and here this girl screaming for help. Apparently, she kneed her testicular cancer bf in the nuts repeatedly.	metaphor	someone else
	memorial find. 10% of your bills went to leukemia and lymphoma research. when amber was around she brightened everyone’s day in one way.	viewpoint	someone else

## Methods

### System Pipeline

**Figure 1:**
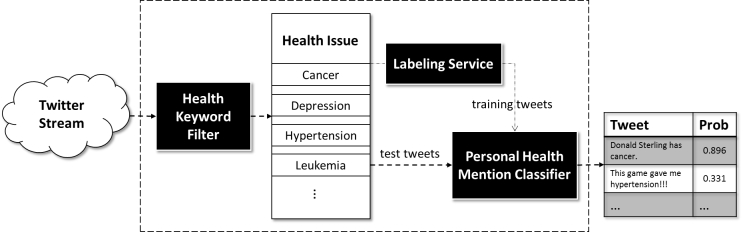
Framework for personal health mention detection over Twitter. First, tweets are filtered into bins according to health issue topic. A portion of the tweets are supplied to a labeling service. The labeled data is then applied to train a classifier to detect personal health mentions.

Figure 1 provides a high-level summary of the system engineered to detect personal health mentions on Twitter. The system is composed of three primary components: (1) a filtering service (eg, a keyword filter based on health issues), (2) a labeling service, and (3) a health mention classification service. First, tweets collected via the Twitter streaming API are passed into a filter and stored in a bin indicative of a specific health issue. Next, a sample of the tweets associated with these health issues are sent to a labeling service (eg, MT). Once labeling is complete, a personal health mention classifier is trained and applied to report the probability that new incoming tweets correspond to such mentions.

### Construction of a Health Mention Corpus

**Figure 2:**
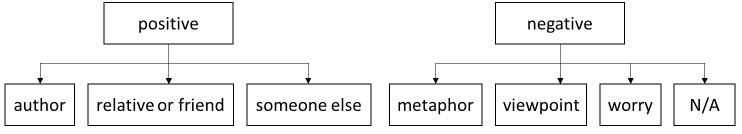
Label hierarchy.

To create a labeled corpus of health status mentions, we solicited annotators through MT. Specifically, we set up a survey for labeling a corpus on MT, the details of which are in Multimedia Appendix 1. For each tweet, we directed two MT masters to select the best of seven options that describe how the tweet uses the health issue. These options represent the common usage of most health issues. We validated the reliability of the MT masters by illustrating that they exhibit high concordance in their labels (details in Tables A-2, A-3 in Multimedia Appendix 1, and in Multimedia Appendix 2). Figure 2 depicts how the options relate to the positive and negative labels.

The positive class includes the labels of author, relative or friend, and someone else. The negative class consists of labels for metaphor, viewpoint, and worry. Table 1 provides examples of tweets and the labels supplied by the MT masters. The last option label, N/A, which means none of the above, is also treated as a negative label in this investigation because it was observed (by the authors) that such labels were generally negative. For instance, these include tweets with job related information, which is spam that has nothing to do with a personal health mention.

For the purposes of this study, we created four types of datasets. The formalization of the design of these datasets is available in Table B-1 in Multimedia Appendix 3. We refer to the first as the gold standard dataset. It consists of all tweets with labels agreeing at the positive (negative) level. This dataset represents an ideal case where readers can determine when a tweet communicates personal health status. For example, this dataset treats tweets as positive when labeled as author by one MT master and someone else by a second MT master. By contrast, this dataset discards tweets labeled as relative or friend and worry.

Given the difficulty in labeling tweets in practice, we generated three additional datasets to resolve label conflicts. The first is the conflict as positive (CAP) dataset, which treats tweets with conflicting labels as positive. The second is the conflict as negative (CAN) dataset, which treats tweets with conflicting labels as negative. The third is the TieBreak dataset, which uses a third MT master to break the tie. These datasets represent the best case, the worst case, and the general case in the real world and we rely upon them to assess the system’s scalability.

### System Classifier Evaluation Roadmap

System scalability emphasizes the ability to detect mentions for many, potentially unknown, health issues communicated via social media, using the labeled tweets from a limited number of health issues.

To formalize the scenario, let *D* be the set of health issues and *X* and *Y* be the set of health issues selected to train and test the classifier, respectively. By default, *X, Y* ⊆ *D*.

**Figure 3:**
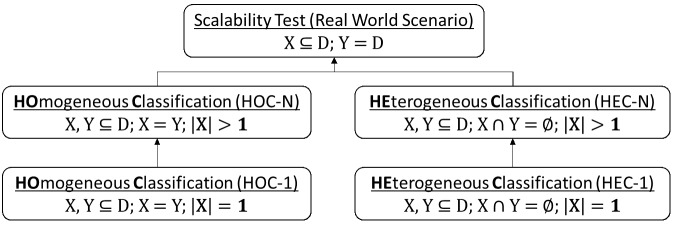
Overview of evaluation strategies for the personal health status mention classifier. Note, D={d1, d2, …, dn} is set of health issues, X is set of health issues selected to train classifier, and Y is set of health issues used to test classifier.

As depicted in Figure 3, we assess two variations on classification. The first, which we refer to as homogeneous classification, corresponds to the traditional machine learning setting where a classifier is trained and tested on tweets from the same health issue. The second, which we refer to as heterogeneous classification, corresponds to when we train and test the classifier on tweets from disparate health issues. This type of scenario arises when a researcher attempts to reuse a classifier developed for one health issue on a different problem. Figure 3 further illustrates two training strategies to scale the system in a real-world scenario: train the classifier on tweets from (1) one health issue, which results in homogeneous classification with |*X*| = 1 (HOC-1) and heterogeneous classification with |*X*| = 1 (HEC-1), and (2) many health issues, which results in homogeneous classification with |*X*| > 1 (HOC-N) and heterogeneous classification with |*X*| > 1 (HEC-N).

The ideal scalability test is to train an HOC-1 classifier for every health issue in *D* with a sufficient quantity of labeled tweets. However, it is difficult to realize this scenario in practice because of limited budgets for gathering and annotating such corpora. As such, we performed a series of experiments to compare the performance of the various models (ie, HOC-1, HOC-N, HEC-1, and HEC-N) and leverage the best model to conduct scalability tests in a real-world scenario.

### Performance Measures

**Figure 4:**
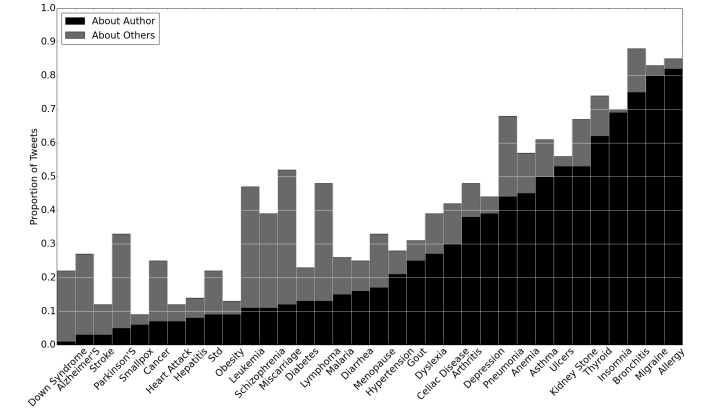
The extent to which people tweet about themselves versus others when disclosing personal health status. Note that this is a stacked bar chart, such that the sum of the author and others proportions corresponds to the overall proportion of positive instances.

To assess the performance of the system, we rely upon the standard measures of precision and recall. In our setting, precision (P) corresponds to the proportion of tweets classified as positive that are in fact positive. Recall (R)corresponds to the fraction of real positive tweets that are classified as positive. Given the large volume of tweets and the often unbalanced positive/negative class ratio per health issue (see Table 2 and Figure 4), we emphasize P while setting R to a reasonable level. Henceforth, we report the area under the PR curve (AUPRC) to evaluate how a classifier performs in general. We consider the PR curve, which can be more indicative of a classifier’s performance when the class ratio is highly imbalanced [40]. To characterize general performance, we report on AUPRC when testing the scalability of the system.

### Health Status Classifier

One of the aims in this research is to examine whether we can use classifiers trained with tweets from multiple health issues to detect personal health mentions about other health issues. Hence, it should be noted that the goal of our research is to examine the effectiveness of classifiers when supplied with a set of known (or off-the-shelf) features. We use a Multinomial Naïve Bayes (MNB) binary classifier based on four types of features associated with tweets. Alternatively, we can plug other learning algorithms, such as logistic regression or a support vector machine, into the framework as the base classifier. Previous investigations verified the effectiveness of such features [33,34,37-39].

- Nouns, verbs, and pronouns. We transformed each word into its lemma form. Though pronouns are often defined as stop terms (which are discarded in traditional natural language processing), they are retained because they can disclose the personal health status of a friend or family member (eg, “My mom makes having cancer look good”).
- Dependencies. These are grammatical relations [41] between words in a tweet, such that one of the words is a health issue. We replaced terms for health issues with the keyword diagnosis to compact the feature space. For example, the dependency (“dobj”, “have”, “cancer”) is converted into a feature that can be supplied to MNB, dobj_have_diagnosis.
- Punctuation and Emoji. These can indicate an author’s emotion and may improve classification (e.g., “my uncle is cancer free !!!!!! lol”).
- HTTP LINK, #hashtags, and @username. These features represent the existence of link, hashtag, and @username in a tweet, respectively.

### Experiment Design

#### Overview

In our experiments, we highlight the evaluation of two important factors that can affect the scalability of a classifier: (1) the diversity of health issues in the training data, and (2) the quantity of training tweets. When we compare different classifiers, we focus on the former. When we test system scalability, beside the system scalability, we also evaluate the performance of the classifiers with different size of training dataset. The following provides details of the experiment design.

#### Dataset

We use the 34 health issues depicted in Figure 4 to represent D and define a synthetic health issue, or SYND, as the union of cancer, depression, hypertension, and leukemia. We select cancer and leukemia, for which tweets are skewed toward communicating about other people’s health status, and depression and hypertension, for which tweets are skewed toward communicating about the author’s health status. We first applied the keywords (shown in Table D-1 in Multimedia Appendix 4), which were selected based on these health issues under the guidance of a clinical expert, to filter for tweets associated with the keywords. Then, we chose 1000 tweets, at random, for each of the four health issues to obtain the gold standard datasets. We also choose 100 tweets, at random, for each of health issue in D to generate gold standard, CAN, CAP and TieBreak datasets.

#### Comparison Between HOC-1 and HOC-N

We use the cancer, depression, hypertension, and leukemia gold standard datasets to train each homogeneous classifier. There are two situations where we can evaluate how the diversity of health issues in the training data influence the homogeneous classifiers. First, suppose that we aim to detect multiple health issues. Given a fixed number of training tweets, how does an HOC-N classifier (eg, trained with SYND) differ from a group of HOC-1 classifiers (eg, four HOC-1 classifiers)? Second, now imagine we wish to perform detection for only one single health issue (eg, cancer). Given a fixed number of training tweets, how does a HOC-N classifier (eg, trained with SYND and test on cancer) differ from the associated HOC-1 classifier (eg, cancer HOC-1 classifier)?

#### Comparison Between HEC-1 and HEC-N

To evaluate the diversity of health issues in training dataset, we compare HEC-1 with HEC-N (2 ≤ |*X*| ≤ 4). In particular, we use the cancer, depression, hypertension and leukemia gold standard datasets for training and the gold standard dataset of D SYND to test all of the heterogeneous classifiers.

#### System Scalability Test

When assessing system scalability, we test the classifier on the CAN, CAP, and TieBreak datasets of D. This enables the evaluation of the performance of the system in a real-world scenario. We also test the classifier trained with different number of tweets.

#### Experimental Methodology

For each experiment, we stratify the tweets and generate 30 train-test sets. In doing so, (1) each set preserves the proportion of samples for each positive (negative) class, and (2) the data is partitioned, such that we train on 80% of the tweets while we test on the remaining 20%. To control the comparison, the size of the training set for each compared classifier is equivalent.

## Results

### Dataset

We used the Twitter streaming API to filter for tweets between May 7, 2014 and July 23, 2014 that were (1) published in the contiguous United States according to their geolocation, and (2) written in the English language only. A total of 261,468,446 tweets were subject to a filter composed of keywords for 34 health issues, resulting in 281,357 tweets (0.11%) for further investigation.

### How People Disclose Personal Health Status on Twitter

To demonstrate the opportunities for a personal health mention detection system, we conducted an investigation to test H1, H2, and H3. We chose 100 tweets, at random, for each of the 34 health issues as shown along the x-axis of Figure 4, to generate the TieBreak dataset. These health issues are based on common and high impact health issues as defined by the Medical Expenditure Panel Survey [17]. This figure illustrates how often people disclose their own health status as opposed to other individuals’ status. The black bar, “About Author”, represents the proportion of positive tweets with the author label. The gray bar, “About Others”, represents the proportion of positive tweets with the label relative or friends and someone else. For a specific health issue, the sum of the two values is equal to the proportion of positive tweets for this health issue. For example, 40% of the tweets about miscarriages (40/100) disclosed other people’s status, while only 12% (12/100) disclosed the author’s status (such that 52%, 52/100, of the tweets were positive instances).

To test hypothesis H2 (personal health status disclosure rate) and H3 (who the disclosure is about), we define the following null hypotheses: H2_o_: The rate of positive and negative tweets is independent of the health issues, and H3_o_: The rate of tweets disclosing the author’s health status and others’ health status is independent of the health issues.

To test these hypotheses, we used the TieBreak dataset, which (due to randomness) represents 100 samples from each of the 34 distributions regarding how people disclose health status. To test H2, we applied a chi-square test on these two variables: the number of positive tweets and the number of negative tweets in each health issue samples. To test hypothesis H3, we applied a Spearman correlation test on these two variables: the rate of tweets disclosing the author’s health status and the rate of tweets disclosing the others’ health status. We set the alpha level of significance to .05.

The results reveal several notable pieces of evidence, which are related to the first three hypotheses posed above.

- People disclose personal health status on Twitter for a range of health issues (H1). The disclosure rate for each of the 34 health issues is greater than 9%. There are 29 health issues with disclosure rates greater than 20% and 11 health issues with disclosure rates greater than 50%. The latter group includes: allergies (85/100), anemia (57/100), arthritis (48/100), asthma (61/100), bronchitis (88/100), insomnia (70/100), kidney stones (67/100), migraines (83/100), miscarriages (52/100), pneumonia (68/100), thyroid (74/100) problems, and ulcers (56/100).
- Health status disclosure rate is dependent on the health issue, χ^2^_33_=697, *P*<.001. For instance, more than 80% of the tweets about migraines (83/100) and allergies (85/100) communicate personal health status. By contrast, only ∼10% of tweets about obesity (13/100) and heart attacks (12/100) communicate personal health status. Bronchitis (88/100) exhibits the largest proportion of tweets that disclose personal health status, while smallpox (9/100) exhibits the smallest proportion.
- The likelihood that people disclose their own versus other people’s health status is dependent on the health issue, *Z*=−5.745, *P*<.001. For instance, 69% (69/100) of tweets about insomnia disclose the author’s personal health statuses compared, while only 1% (1/100) disclose another person’s status. By contrast, 1% (1/100) of the tweets for Down syndrome disclose the author’s status, while 21% (21/100) disclose another person’s status.

### Classification Evaluation

#### Classification Data Set

We extracted the gold standard datasets for each of the four health issues mentioned in the Methods section. Table 2 summarizes the number of tweets in each class. Except leukemia, which has a balanced positive and negative instance space, there were substantially more negative than positive tweets. Due to the definition of SYND, the number of positive and negative tweets of the synthetic health issue is the sum of the four health issues.

**Table 2:** The number of positive and negative tweets in the gold standard datasets.

Tweet	Cancer	Depression	Hypertension	Leukemia	SYND^a^
Positive	166	261	211	436	1074
Negative	697	461	551	423	2132

^a^SYND: synthetic health issue (D).

#### Most Informative Features

Before conducting an in-depth empirical investigation, we inspected the classifiers and their corresponding features to determine if they are intuitive. Here, we report on the top 10 informative features by training in a homogeneous classification setting with tweets of each of the five health issues (cancer, depression, hypertension, leukemia, and SYND). Table 3 reports these features for each classifier.

The results show the effectiveness of feature selection in several ways. First, more than five features are pronouns, such as I, my, and she (which was also confirmed in [32]). These are stop words that are typically removed in the context of general text classification. However, in our scenario, they appear to signify users who disclose health information about themselves and others (eg, “my mom makes having cancer look easy”). Second, certain words, such as get, have, and battle, when applied in conjunction with a health issue, can disclose personal health status (eg, “my friend lost his battle to leukemia”). Third, dependencies, such as “obj_have_diagnosis”, are strong positive indicators (eg, “I have seasonal allergy”).

This table also provides several notable results about other behaviors when people disclose personal health status. For instance, people often include @someone in health mentions. They use links to provide additional information such as pictures, locations, or texts, or use exclamation mark to express strong feelings about personal health status.

The hypertension classifier was notable because it had specific health-related terminology ranked highly. Specifically, the term blood is highly informative for this classifier. We suspect this is because hypertension is commonly referred as high blood pressure.

**Table 3:** The most informative features for homogeneous health mention classification.

Rank	Cancer	Depression	Hypertension	Leukemia	SYND^a^
1	I	I	I	I	I
2	my	my	my	My	My
3	!		have		
4		you		HTTP LINK	!
5	you	it	dobj_have_diagnosis	!	Have
6	have	go	!	She	HTTP LINK
7	she	poss_diagnosis_my	get	Have	She
8	He	!	she	He	You
9	HTTP LINK	get	it	Battle	obj­_have_diagnosis
10	obj_have_diagnosis	have	blood	Help	He

^a^SYND: synthetic health issue (D).

#### Homogeneous and Heterogeneous Classification

In this experiment, we compared the effectiveness of homogeneous and heterogeneous classifiers and then testing on tweets from each of the five health issues. Table 4 provides the AUPRCs for each homogeneous (along the diagonal) and heterogeneous (off diagonal cells) health mention classifier. Each row corresponds to the health issue relied upon for training the classifier, while each column corresponds to the health issue the classifier was applied to. To test the significance, we ran a *t* test when the results followed a normal distribution and a Kolmogorov-Smirnov (KS) test otherwise.

First, it should be noted that each homogeneous classifier outperforms the heterogeneous classifiers when testing the corresponding health issue tweets, but such classifiers do not generalize. It can be seen that the leukemia HOC-1 classifier achieved the highest AUPRC. This may be due to the balance in the positive and negative classes for this health issue. However, it was observed that the homogeneous classifiers exhibited much higher variance compared to the heterogeneous classifiers. This suggests that heterogeneous classifiers may yield stable results.

Second, the HEC-1 classifier may tend to obtain a better AUPRC when testing on health issues with a similar author-to-others disclosure rate. For instance, cancer achieved the best AUPRC when testing on leukemia tweets. Meanwhile, leukemia achieved the best AUPRC when testing on cancer tweets. Depression and hypertension also achieved the best AUPRC when testing on each other.

Third, it also shows that SYND heterogeneous classifier (HEC-N) was the second best heterogeneous classifier when testing on cancer, depression, and leukemia tweets, and the best heterogeneous classifier when testing on hypertension. Considering that the HEC-1 classifier is specialized to a certain health issue, the HEC-N classifier may provide a more scalable alternative when filtering for personal health mentions on other health issues.

**Table 4:** AUPRC for homogeneous and heterogeneous classifiers.^a^

	Cancer	Depression	Hypertension	Leukemia	SYND
	mean (SD)				
Cancer	0.732 (0.058)	0.528 (0.018) ^b^	0.552 (0.014)^b^	0.869 (0.009)^b^	0.728 (0.009)^b^
Depression	0.441 (0.007)^b^	0.663 (0.054)	0.611 (0.014)^b^	0.821 (0.006)^b^	0.666 (0.006)^b^
Hypertension	0.451 (0.009)^b^	0.646 (0.011)	0.664 (0.062)	0.726 (0.008)^b^	0.616 (0.006)^b^
Leukemia	0.638 (0.011)^b^	0.603 (0.011)^b^	0.559 (0.019)^e^	0.936 (0.019)	0.579 (0.007)^b^
SYND^f^	0.625 (0.022)^e^	0.618 (0.026)^d^	0.626 (0.019)^c^	0.831 (0.023)^b^	0.820 (0.0180

^a^ AUPRC: area under the precision recall curve. Classifiers were trained with row health issue tweets and tested on column health issue tweets. Within each column, a hypothesis test was conducted between HOC-1 and each model that is not HOC-1 (eg, HOC-1 vs HEC-1).

^b^ *P*<.001

^c^ *P*=.002

^d^ *P*=.003

^e^ *P*=.004

^f^SYND: synthetic health issue (D).

**Table 5:** AUPRC of homogeneous health mention classifiers, given the same number of training tweets.^a^

Classifier	Cancer	Depression	Hypertension	Leukemia
	mean (SD)			
HOC-1^b^	0.732 (0.058)	0.663 (0.054)	0.664 (0.063)	0.936 (0.019)
HOC-N^c^	0.723 (0.061)	0.645 (0.053)	0.672 (0.070)	0.927 (0.022)
HOC-N‡	0.756 (0.050)	0.681 (0.050)	0.702 (0.059)^d^	0.940 (0.021)

^a^AUPRC: area under the precision recall curve. Within each column, the hypothesis test was conducted between HOC-1 and each model that is not HOC-1 (eg, HOC-1 vs HOC-N).

^b^HOC-1: homogeneous classification with |X| = 1

^c^HOC-N: homogeneous classification with |X| > 1

^d^ *P*=.015

#### Comparison of Homogeneous Classifiers

In this experiment, we evaluated how homogeneous classifiers are influenced by (1) the number of health issues in the training set, and (2) the number of tweets used for training classifiers. Table 5 shows the results for the HOC-1 and HOC-N classifiers when testing on the tweets of each health issue. For each column, we trained homogeneous classifiers HOC-1 and HOC-N with the same number of training tweets. The number of training tweets for HOC-N^‡^ classifier equaled to the number of all the tweets training for each HOC-1 classifier. HOC-N^‡^ is introduced to compare classifiers in a scenario often encountered in practice. For instance, imagine there is a fixed budget (eg, monetary quantity) through which we can only label 2000 tweets. If we have four HOC-1 classifiers, then we can only allocate 500 tweets to each. However, we can allocate all 2000 tweets to the HOC-N classifier. Again, we ran a *t* test when the results failed to followed a normal distribution and a KS-test otherwise.

The hypothesis tests showed that only the HOC-1 and HOC-N^‡^ classifiers are statistically significant when testing on hypertension tweets (*P*=.015). This suggests that HOC-N classifiers are expected to have similar performance with HOC-1 classifiers when each classifier is trained with the same number of training tweets. However, if the total number of training tweets is fixed, the HOC-N classifier will outperform the combination of HOC-1 classifiers.

This indicates that the HOC-N classifier can serve as a substitute for HOC-1 classifiers.

#### Comparison Between Heterogeneous Classifiers

**Figure 5:**
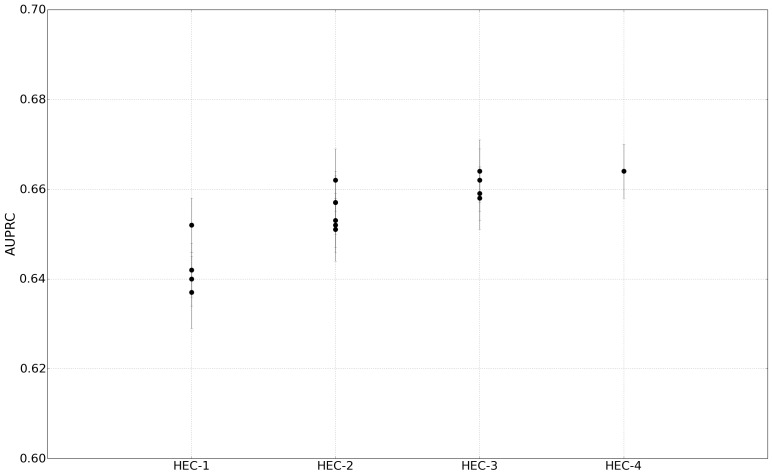
Comparison Between heterogeneous classifiers HEC-1 and HEC-N trained on cancer, depression, hypertension, and leukemia, and tested on the remaining 30 health issues. The tweets of each test health issue stratified with respect to their rate of observation.

In this experiment, we evaluated how the number of health issues in the training set influence the heterogeneous classifiers. Figure 5 shows the results of HEC-1 and HEC-N (N ∈ {2, 3, 4}) when testing on the other 30 health issues. For HEC-1, it should be noted that the cancer HEC-1 achieved the best AUPRC. This may stem from the fact that cancer can be invoked to communicate a wide variety of concepts beyond an individual’s health status, such as the Zodiac, the name of a physical building, or a metaphor. The results also indicate that HEC-N tends to outperform HEC-1.

This suggests hypothesis H4 may be true, provided the classifier is based on an appropriate mixture of health issues. However, determining an optimized group of health issues to achieve an HEC-N classifier with performance comparable to HEC-1 classifier is left to future investigation.

Based on these findings, we use HOC-N and HEC-N to conduct the system scalability test.

### System Scalability

After breaking ties, 43.7% of the TieBreak dataset are positive instances. Based on this proportion, there are approximately 120,260 positive instances out of 281,357 tweets in the health issue bins (or 0.046% of all the collected tweets). Table 6 reports the distribution of positive and negative tweets in each dataset.

**Figure 6:**
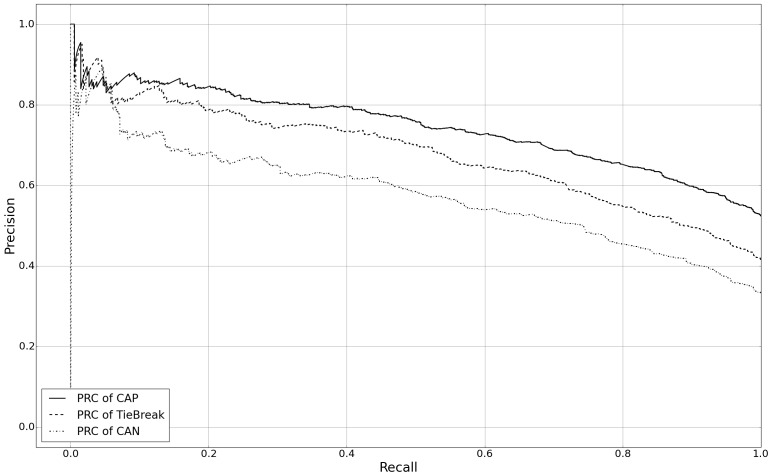
PR (precision recall) curves for testing on the gold, CAN (conflict as negative), and CAP (conflict as positive) datasets.

We trained the SYND classifier with the gold standard datasets for cancer, depression, hypertension, and leukemia, and tested it on the other three types of datasets. Figure 6 depicts the PR curves for each dataset and shows the average and standard deviation of AUPRC. The upper line corresponds to testing on the CAP dataset (AUPRC 0.753, SD 0.005), the middle line corresponds to testing on the TieBreak dataset (AUPRC 0.685, SD 0.005) and the lower line corresponds to testing on the CAP dataset (AUPRC 0.594, SD 0.007). When fixing the recall to 0.4, it was observed that the CAP, TieBreak, and CAN scenarios yield a precision of 0.8, 0.77, and 0.61, respectively. These results demonstrate the scalability of the system classifiers to obtain a high precision with a reasonable recall when testing many other health issues in the Twitter environment.

**Figure 7:**
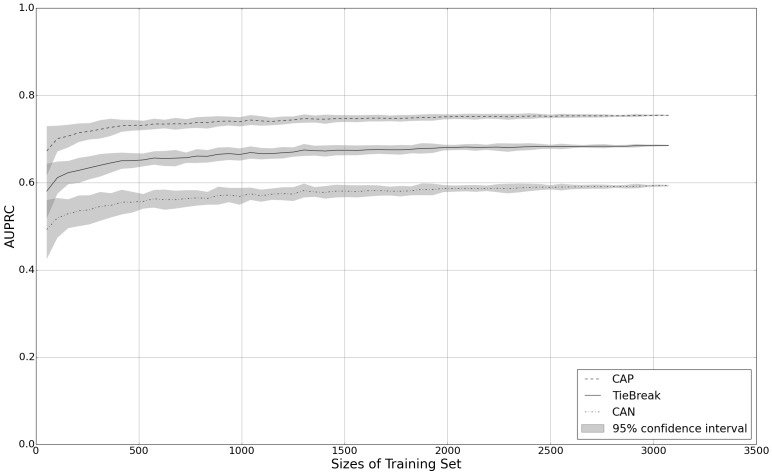
Performance of the SYND (synthetic health issue) classifier with a varying amount of training data.

Figure 7 shows how the size of the training set influences the AUPRC of the classifiers. For each training set, the mean AUPRC and a 95% confidence interval is illustrated in the gray area. For each dataset, the results suggest that AUPRC achieves stability when the training set consists of approximately 2000 tweets.

**Table 6:** Class distribution of tweets in the datasets.

Tweets	Gold	CAN^a^	CAP^b^	TieBreak
Positives	1082	1082	1718	1366
Negatives	1539	2175	1539	1891

^a^CAN: conflict as negative

^b^CAP: conflict as positive

## Discussion

### Principal Findings

There are several notable findings from this investigation. First, Twitter users disclose the health status of themselves and others. Second, the health status disclosure rate may depend on the health issue. Third, how people disclose their own and other people’s health status may also be health issue dependent. Fourth, tweets related with a small group of health issues can train a scalable classifier to detect health mentions on Twitter streams.

Another interesting phenomenon illustrated from the PR curves (Figure 6) is that the system classifier, trained with the tweets for which MT masters exhibited high concordance in their labels, is more likely than MT masters to classify tweets with conflict labels as positive. One possible explanation is that the classifier makes its decision based on thousands of examples, while most MT masters made decisions only with the description of the survey, which indicates that the classifier may be more familiar with the labeling task. This suggests there may be a difference between using an expert and crowdsourcing to generate the labeled corpus. However, determining how to best leverage the crowd to mimic an expert is beyond the scope of this investigation.

### Impact on Health Related Research

According to our investigation, roughly 44% of the tweets containing health issue keywords disclose personal health status. We believe there is a potential for information to assist health care professionals in learning about their patients or their patients’ family medical history, information often missing in the EMRs. This indicates that social media platforms, such as Twitter contains huge amount of personal health care related information that may complement traditional EMRs in research and practice. We recognized that we must still verify the veracity of such data, but an opportunity exists nonetheless.

### Limitations

We wish to highlight several limitations of this investigation. First, two parameters to extract tweets from Twitter streams require configuration: (1) the set of keywords invoked in the filter, and (2) the geolocation applied to discover tweets. Compared to keywords, geolocation can filter tweets disseminated by authoritative organizations (due to the absence of “coordinates” and “place” information in these tweets), such as the American Cancer Society, and thus greatly reduce noise. However, it should be noted that invoking such a filter can also exclude the tweets of individuals who choose not to disclose their location. A second limitation exists in the survey provided to the MT masters for labeling the corpus. Specifically, we assumed the N/A option was a member of the negative class, but this could be an incorrect assumption in certain instances. Third, this investigation was restricted to only 34 health-related phenomena, which is clearly only a sample of all possible health issues. The keywords filter service can be enhanced by integrating a laymen health vocabulary [42]. Given that this study shows there is (1) high variability in the rate at which people tweet about a certain health issue, and (2) to whom the statement of health issue corresponds, it will be critical to investigate how these methods fare in the context of other health issues.

### Conclusions

Recent studies demonstrate the information communicated through social media platforms, such as Twitter and Facebook, could supplement traditional medical and epidemiological research. In this paper, we showed that a health mention detection system can be designed and deployed for microblogging systems, such as Twitter. At the same time, we illustrated that the information communicated through such mentions can disclose the health status of the authors and other individuals at a wide range of rates. Our experimental investigation further showed that the combination of tweets from several health issues can yield a classifier that dominates a classifier based on the tweets of a single health issue. This may enable the system to use a small amount of training data to build a classifier that detects health status mentions across a range of health issues. We envision several opportunities for extending this work. First, we believe the scalability of the classifier may be improved by determining the minimal set of health issues and features (eg, more complicated grammar features). Second, we anticipate that the performance of the classifier could be improved be accounting for context, such as dialogue, relationships in the network, and profile information as new supplemental features. Finally, while the rate that health status is disclosed for the author versus other individuals is dependent upon the considered health issue, further investigation is required to determine what drives this disparity. We suspect, for instance, that it may be dependent on the sensitivity and severity of health issues, but this is only a conjecture.

## Appendix

Multimedia Appendix 1 Example question posed to Mechanical Turk masters.

Multimedia Appendix 2 Concordance between the system classifier and Mechanical Turk masters.

Multimedia Appendix 3 Summary of four datasets.

Multimedia Appendix 4 Keywords used to filter tweets.

## Abbreviations

AUPRC: area under the precision recall curve
CAP: conflict as positive
CAN: conflict as negative
EMR: electronic medical record
HEC-1: heterogeneous classification with |X| = 1
HEC-N: heterogeneous classification with |X| > 1
HOC-1: homogeneous classification with |X| = 1
HOC-N: homogeneous classification with |X| > 1
KS: Kolmogorov-Smirnov Test
MNB: Multinomial Naïve Bayes
MT: Mechanical Turk
N/A: none of the above
SYND: synthetic health issue
