# Supplementary material for: A Scalable Framework to Detect Personal Health Mentions on Twitter
Source: J Med Internet Res. 2015 Jun 5;17(6):e138. doi: 10.2196/jmir.4305 (PMC4526910; doi:10.2196/jmir.4305)
Supplement: Multimedia Appendix 1 [file jmir_v17i6e138_app1.pdf]

Multimedia Appendix 1

The following provides an example question posed to the MT masters.

How is health issue information used in the tweet?
"Lauren Hill is at it again! The bball hero with cancer scored in her teams first home game. http://go.fox59.com/1yTnkTh"

1. The tweet discloses the health status of the author. (e.g., going to get my last chemo treatment)
2. The tweet discloses the health status of the author's family members or friends. (e.g., my uncle just found out he has cancer; my friend Tom has been cancer free for four years)
3. The tweet discloses the health status of someone else, excluding the author, the author's family members and friends. (e.g., She has hypertension; Donald Sterling Is Battling Cancer)
4. The tweet uses the health issue as a metaphor (e.g., he is a cancer; the game makes me high blood pressure)
5. The tweet expresses a viewpoint on the health issue, or some kind of support to general patients with the health issue (excluding those specific persons mentioned in option 1, 2 and 3). (e.g., I think cancer is horrible; Guys I'm #feelingnuts raising awareness for testicular cancer I'm nominating; Breast Cancer Awareness Month is JUST around the corner)
6. The tweet expresses a worry about the health issue. (e.g., I hope I don't get cancer by using my cell phone)
7. None of the above

We launched three surveys on MT to obtain the labeled datasets: 1) labeling 1000 tweets for each of the four health issues (see Method Section), two MT masters per tweet, 2) labeling 100 tweets for each of the thirty four health issues (see Figure 4), two MT masters per tweet, and 3 breaking tie for those tweets with conflict labels in the second survey, one MT master per tweet. Table 7 shows the summary on the MT workers for each survey:

Table A-1: Summary of labeling tasks on MT.

Survey	Number of Masters	Mean of Labeled Tweets	St.Dev
4 Health Issues	144	27.8	42.1
34 Health Issues	65	52.8	85.1
Tie Break	22	62.8	137.3

Table A-2 and Table A-3 show the strength of agreement for the two labeling tasks in MT, based on Kappa scores, which confirmed the reliability of the MT masters' tasks.

Table A-2: Strength of agreement in labeling 34 Health Issues (100 tweets for each health issue)

Strength	Good (0.61-0.80)	Moderate (0.41-0.60)	Fair (0.21-.0.40)	Poor (< 0.20)
# health issues	15	14	5	1

Table A-3: Strength of agreement for labeling 4 Health Issues (1000 tweets for each health issue)

Strength	Good (0.61-0.80)	Moderate (0.41-0.60)	Fair (0.21-.0.40)	Poor (< 0.20)
# health issues	2	2	0	0
