# Supplementary material for: A Scalable Framework to Detect Personal Health Mentions on Twitter
Source: J Med Internet Res. 2015 Jun 5;17(6):e138. doi: 10.2196/jmir.4305 (PMC4526910; doi:10.2196/jmir.4305)
Supplement: Multimedia Appendix 2 [file jmir_v17i6e138_app2.pdf]

Multimedia Appendix 3

To assess the concordance between the system classifier and MT masters, we computed the positive concordance rate as follows:

$$\frac{\#\text{positive tweets with "11" label}}{\#\text{positive tweets with "11" label} + \#\text{positive tweets with "101" or "011" label}}$$

We computed this proportion as a function of the threshold after conducting HEC-N classification on 34 health issues. Figure C-1 provides a summary of the results.
It is clear that there is a positive correlation between the concordance and the threshold. Specifically, a linear regression of these concepts years an R^2 of 0.734. We note that the change in concordance proportion grows slowly, starting at 0.8 at a threshold close to 0 and increasing to 0.86 by a threshold of 0.95. As a result, it appears that our classifier agrees with the increasing certainty of our MT pool. We believe this provides further justification in the reliability of our MT masters.

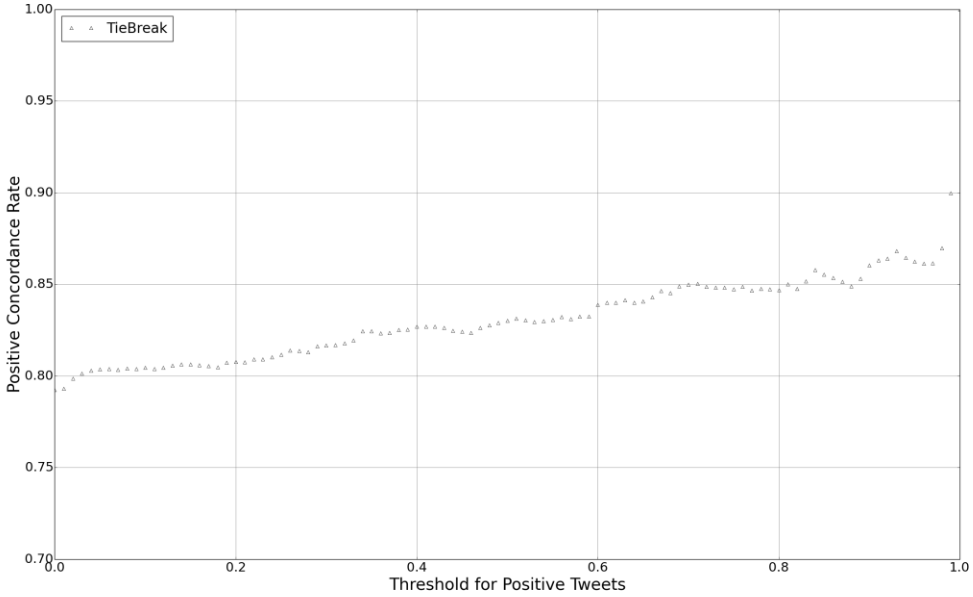

Figure 8: Concordance between the system classifier and MT masters.
