# Supplementary material for: A Scalable Framework to Detect Personal Health Mentions on Twitter
Source: J Med Internet Res. 2015 Jun 5;17(6):e138. doi: 10.2196/jmir.4305 (PMC4526910; doi:10.2196/jmir.4305)
Supplement: Multimedia Appendix 3 [file jmir_v17i6e138_app3.pdf]

Multimedia Appendix 2

Table B-1: Summary of the four datasets investigated in this study, where 0 and 1 correspond to a negative and positive label, respectively.

Dataset	Master 1	Master 2	Master 3	Label
Gold Standard	0	0	-	0
	1	1	-	1
Conflict as	0	0	-	0
	0	1	-	1
	1	1	-	1
Conflict as	0	0	-	0
	0	1	-	0
	1	1	-	1
TieBreak	0	0	-	0
	1	1	-	1
	0	1	0	0
	0	1	1	1

Table B-1 summarizes the four types of datasets studied in this work: 1) the gold standard, 2) the conflict as positive (CAP), 3) the conflict as negative (CAN), and 4) the TieBreak.
