# Supplementary material for: A Scalable Framework to Detect Personal Health Mentions on Twitter
Source: J Med Internet Res. 2015 Jun 5;17(6):e138. doi: 10.2196/jmir.4305 (PMC4526910; doi:10.2196/jmir.4305)
Supplement: Multimedia Appendix 4 [file jmir_v17i6e138_app4.pdf]

Multimedia Appendix 4

Table D-1: Keywords used to filter the related tweets.

<table>
<tr><th>Health Issues</th><th>Keywords</th></tr>
<tr><td>asthma</td><td>asthma|#asthma</td></tr>
<tr><td>anemia</td><td>anemia|#anemia</td></tr>
<tr><td>arthritis</td><td>arthritis|#arthritis</td></tr>
<tr><td>allergy|alleries</td><td>allergy|#allergy|allergies|#allergies</td></tr>
<tr><td>alzheimer's</td><td>alzheimer's|#alzheimer's</td></tr>
<tr><td>bronchitis</td><td>bronchitis|#bronchitis</td></tr>
<tr><td>bursitis</td><td>bursitis|#bursitis</td></tr>
<tr><td>cancer</td><td>cancer|#cancer</td></tr>
<tr><td>celiac disease</td><td>celiac disease|#celiac diesase</td></tr>
<tr><td>depression</td><td>depression|#depression</td></tr>
<tr><td>diabetes|high blood sugar</td><td>diabetes|#diabetes|high blood sugar|#high blood sugar</td></tr>
<tr><td>diarrhea</td><td>diarrhea|#diarrhea</td></tr>
<tr><td>down syndrome</td><td>down syndrome|#down syndrome</td></tr>
<tr><td>dyslexia</td><td>dyslexia|#dyslexia</td></tr>
<tr><td>gout</td><td>gout|#gout</td></tr>
<tr><td>heart attack</td><td>heart attack| #heart attack</td></tr>
<tr><td>hepatitis</td><td>hepatitis|#hepatitis</td></tr>
<tr><td>hypertension|high blood pressure</td><td>#hypertension|#high blood pressure</td></tr>
<tr><td>insomnia</td><td>insomnia|#insomnia</td></tr>
<tr><td>leukemia</td><td>leukemia|#leukemia</td></tr>
<tr><td>lymphoma</td><td>lymphoma|#lymphoma</td></tr>
<tr><td>malaria</td><td>malaria|#malaria</td></tr>
<tr><td>menopause</td><td>menopause|#menopause</td></tr>
<tr><td>migraine</td><td>migraine|#migraine</td></tr>
<tr><td>miscarriage</td><td>miscarriage|#miscarriage</td></tr>
<tr><td>obesity</td><td>obesity|#obseity</td></tr>
<tr><td>parkinson's</td><td>parkinson's|#parkinson's</td></tr>
<tr><td>pneumonia</td><td>pneumonia|#pneumonia</td></tr>
<tr><td>schizophrenia</td><td>schizophrenia|#schizophrenia</td></tr>
<tr><td>smallpox</td><td>smallpox|#smallpox</td></tr>
<tr><td>std|stds</td><td>#std|#stds</td></tr>
<tr><td>stroke</td><td>stroke|#stroke</td></tr>
<tr><td>thyroid</td><td>thyroid|#thyroid</td></tr>
<tr><td>ulcers</td><td>ulcers|#ulcers</td></tr>
</table>
